# Metal Ions Bound to Prion Protein Affect its Interaction with Plasminogen Activation System

**DOI:** 10.1007/s10930-021-10035-4

**Published:** 2022-01-17

**Authors:** Maryam Borumand, Vincent Ellis

**Affiliations:** 1 University of East Anglia, Norwich Research Park, Norwich, NR4 7TJ UK; 2 University of Buckingham, Hunter Street, Buckingham, MK18 1EG UK

**Keywords:** Plasminogen, Tissue plasminogen activator (tPA), Plasmin, Plasminogen regulation, Prion, Prion disease

## Abstract

Prion diseases are a group of neurodegenerative diseases, which can progress rapidly. Previous data have demonstrated that prion protein (PrP) stimulates activation of plasminogen (Plg) by tissue plasminogen activator (tPA). In this study, using spectroscopic method, we aimed to determine whether PrP’s role in activating Plg is influenced by metal binding. We also investigated the region in PrP involved in binding to tPA and Plg, and whether PrP in fibrillar form behaves the same way as PrP unbound to any metal ion i.e., apo-PrP. We investigated the effect of recombinant mouse PrP (residues 23-231) refolded with nickel, manganese, copper, and a variant devoid of any metal ions, on tPA-catalyzed Plg activation. Using mutant PrP (H95A, H110A), we also investigated whether histidine residues outside the octarepeat region in PrP, which is known to bind tPA and Plg, are also involved in their binding. We demonstrated that apo-PrP is most effective at stimulating Plg. PrP refolded with nickle or manganese behave similar to apo-PrP, and PrP refolded with copper is least effective. The mutant form of PrP did not stimulate Plg activation to the same degree as apo-PrP indicating that the histidine residues outside the octarepeat region are also involved in binding to tPA and Plg. Similarly, the fibrillar form of PrP was ineffective at stimulating Plg activation. Our data suggest that upon loss of copper specifically, a structural rearrangement of PrP occurs that exposes binding sites to Plg and tPA, enhancing the stimulation of Plg activation.

## Significance Statement

Proteins, such as prion protein, are an important part of cell function. Changes in structure and function of the protein can disrupt normal cellular function, as well as cause a buildup of protein aggregates. This is seen in prion diseases. This research demonstrates that simply looking at prion protein alone is not sufficient in determining molecular basis of the prion diseases. Understanding interaction of prion protein with important proteases like the plasminogen action system, could enable us to differentiate the normal form of PrP; PrP^C^, from the disease form; PrP^Sc^. A further highlight of this research is that metal ions can play a key role in neurodegenerative diseases and so need to be considered.

## Introduction

The Plg activation system, which consist of Plg, tPA and urokinase Plasminogen Activator (uPA), belong to the family of ‘serine proteinases’ which hydrolyze peptide bonds in proteins. They are synthesized as single chain precursors or zymogens, composed of an N-terminal peptide, followed by various homologous kringle (Kr) structures (each ~ 85 amino acid residues), and a C-terminal serine protease catalytic domain. Four Kr modules in Plg act as lysine binding sites (Miles & Plow, 1985, Stephens et al, 1989), allowing Plg to bind many lysine containing proteins.

Besides the cell surface, non-enzymatic cofactors like fibrinogen, can act to provide a specific template for the Plg activation system. Surface bound Plg is cleaved once at Arg^561^-Val^562^ to produce a trypsin-like serine protease called plasmin, either by tPA (Km=5.9 nM on human umbilical vein endothelial cells (12.7- fold greater efficiency than fluid phase Plg (Hajjar et al, 1986)), or by uPA bound to its receptor (~22-fold acceleration compared to in solution (Ellis et al, 1989)). Subsequently, plasmin cleaves fibrinogen to fibrin, which have been shown to stimulate Plg activation further, leading to large amounts of plasmin being generated (Ellis et al, 1989).

Plasmin occurs in high concentrations in the blood and is widely dispersed in the body. Unlike the Plg activators, which only cleave Plg, plasmin has broad substrate specificity. It cleaves proteins at Lys and Arg residues. As well as fibrinolysis, plasmin is implicated in degrading extracellular matrix (ECM) proteins such as laminin (Lu et al, 2011), cleaving and hence activating metalloproteinases and growth factors. Pericellular proteolysis of ECM molecules alters anchorage, focal adhesion, cytoskeletal structure and signaling; all of which are necessary for cell migration (Parsons et al, 2010).

The exponential process of Plg activation is subject to regulation by endogenous Plg Activator Inhibitor (PAI). Cell-bound active uPA is accessible to inhibition by PAI-1 and PAI-2 (Stephens et al, 1989). The latter is a less efficient inhibitor, found primarily in monocytes and placenta. Conversely, tPA is protected from inhibition by PAI-1 on cell surface (Werner et al, 1999). This indicates that where both Plg activators are present, they may have differential roles in Plg activation.

The Plg activation system is implicated in a wide range of pathologies including cancer (Südhoff & Schneider, 1992). In the neurodegenerative disease Multiple Sclerosis, elevated levels of tPA mRNA have been detected in neurons, in the proximity of areas of demyelination (Teesalu et al, 2002). However, a tPA inhibitor Neuroserpin, has been found to increase following ischaemia in the central nervous system (Yepes & Lawrence, 2004) suggesting cells may use inhibitors as a protective mechanism against perpetual proteinase activity induced by disease or damage to the cell.

In 2000, a study by Fischer et al., linked PrP to Plg activation system. The study showed that Plg binds selectively to the abnormal form of PrP found in disease, referred to as scrapie form; PrP^Sc^. However, Shaked et al., challenged the findings by showing that, detergent combinations that affect membrane microdomains known as rafts, can affect binding of PrP to serum protein, and that at detergent conditions in which rafts are intact, it is the normal PrP isoform; PrP^C^ that binds to blood proteins (Shaked et al., 2002). Later, a study by Cuccioloni et al. (2005), using an optical biosensor, indicated that Plg has a high affinity for both types of PrP. Lysine residues seem to have an important role, since interaction of PrP with Plg was prevented by using poly-L-lysine, and the mutant PrPQ218K had higher binding affinity to Plg than wild type PrP (Ryou et al., 2011, Lee et al., 2017).

When comparing gene expression profiles from brains of scrapie infected mice with normal mice, Xiang et al (2004) found an increase in expression of 121 genes 3-4 months post-inoculation, which included proteins involved in proteolysis and protease inhibition. Even stronger evidence indicating a role for Plg activation system in plaque-forming neurodegenerative diseases comes from studies on Alzheimer’s disease mouse models. Amyloid-beta (Abeta) injected into wild-type mice was rapidly cleared and did not cause neuronal degeneration, whereas Abeta injected into the hippocampus of mice lacking either tPA or Plg persisted, causing neuronal damage (Melchor et al, 2003).

Finding by Ellis et al. (2002), showed that PrP refolded in absence of divalent metal ions can stimulate Plg activation. Rate of Plg activation can be measured rapidly using spectrofluorometry, without any change in nature of the proteins. It provided a new tool for investigating the interaction of Plg with both normal and the disease form of PrP. Interestingly, Ellis et al, (2002) found that the ability of PrP to stimulate Plg activation is reduced when PrP is bound to copper,

PrP is a protein of 254 amino acids, or 209 residues after processing of the amino and carboxyl termini. The protein contains a C-terminal globular domain that extends approximately from residues 121–230. Within the N-terminal (23-120), the octapeptide repeat region, comprised of repeats of the sequence PHGGGWGQ (residues 60-91 in human PrP consist of four His-containing octarepeats, and residues 51-59 consist of the homologous sequence PQGGGGWGQ), is among the most conserved segments of PrP in mammals, which implies a functionally important role. The importance of the octapeptide repeat region of PrP in the pathogenesis of prion diseases is revealed by the fact that insertion of extra peptide repeats within PrP correlates with earlier CJD onset and reduced incubation times in animals (Croes et al., 2004, Castilla et al., 2004).

Techniques such as fluorescence resonance energy transfer, and molecular dynamics simulations, have established that the N-terminal 100 residue domain is unstructured (Gustiananda et al, 2004). On the other hand, a visible absorption band with Circular Dichroism spectra indicated a distinctive structuring of the octarepeat region on Cu (II) binding (Viles et al, 1999). Binding of Cu^2+^ was found to be cooperative and highly pH dependent, suggesting that the binding of Cu^2+^ occurred through association with the histidine residues.

In investigating the connection between the binding of copper to PrP, so far investigators have focused on techniques, which use the properties of PrP such as protease resistance, solubility, conformation, and the ability of the protein to aggregate.

Copper can facilitate the folding of native PrP^C^ into a more protease resistant form (Qin et al., 2000, Quaglio et al., 2001). The necessity to ‘age’ recombinant PrP, however, makes it difficult to determine the conditions required for gain of protease resistance. Indeed, certain anti-PrP^C^ monoclonal antibodies, which showed equivalent intensity of staining of human PrP on fresh cells, showed relative reductions of staining on stored cells, indicating possible structural alterations of PrP^C^ under these conditions (Barclay et al., 1999). Another factor that complicates protease resistance studies is ionic strength. Induction of protease resistance requires a minimum level of ionic strength (Nishina et al., 2004). It is therefore difficult to make any definite conclusions on the role of copper in prion disease based on these properties.

Apart from copper, PrP can also bind zinc, manganese, and nickel (Brown et al., 2000, Jackson et al., 2001, Walter et al., 2007, Brown, 2011), which appear to confer high stability to the protein (Benetti et al., 2014). Disturbances in the levels of divalent metal ions, namely, copper, zinc, and manganese, have been described in prion-infected brain tissues (Wong et al., 2001, Thackray et al., 2002). It has been postulated that binding of these metal ions might modulate the structure and function of PrP^C^, and directly influence its structural conversion to PrP^Sc^ and the formation of amyloid aggregates.

In this study, spectrofluorometry is used to determine whether there is a difference in the way various metal ions affect the function of PrP in stimulating Plg activation. Continuous assays were used in which human Lys^77^-Plg was activated by purified tPA, and the plasmin generated was measured through cleavage of plasmin-specific fluorogenic substrate, VLK-AMC leading to the fluorescent AMC. Lys^77^-Plg, which is more readily activated than native Glu^1^-Plg, and two-chain tPA, the more active form of tPA, were used in this study to prevent possible interference from proteolytic conversion during the assays. In the early phase, the activation reaction is linear so is used to determine the rate of Plg activation, thus allows comparison of rate with different variables.

## Materials and Methods

Institutional ethical approval was not required for this study

### Proteins

Active two-chain tPA was purchased from Boehringer-Ingelheim. Human [Lys^77^] plasminogen (containing lysine at position 77), Glu^1^-plasminogen and purified human plasmin were supplied by Enzyme Research Laboratories. Desafib-X (plasmin-digested fibrinogen, which mimics the effect of fibrin on the activation of plasminogen by t-PA) was purchased from American Diagnostica, Inc. Other chemicals were purchased from Sigma.

### Preparation of Mouse PrP23-231

Mouse PrP was expressed in *Escherichia coli* (BL21) by Professor David Brown’s group (Department of Biochemistry, University of Bath), as described previously (Brown et al., 2000). The recombinant PrP consists of amino acid residues 23-231, constituting full length processed protein, lacking the signal peptide and the GPI-anchor. Furthermore, recombinant PrP is not glycosylated since bacteria do not synthesize enzymes required for glycosylation of proteins. The expressed protein includes a C-terminal polyhistidine tag that allows purification using immobilized nickel-based affinity chromatography. The eluted material was refolded from 8M urea in either deionized water, 1 mM CuSO_4_, or 1 mM NiCl_2_ or MnCl_2_, followed by ultrafiltration and dialysis to remove unbound metal. Note that when PrP refolded in copper is dialyzed in water and kept at − 20 °C or − 80 °C, most of the protein precipitates, therefore PrP containing copper was dialyzed in 10 mM Mes, pH 6.8, to prevent precipitation. PrP mutants H95A and H110A were also expressed and purified by Brown’s lab. The proteins displayed the expected bands on reduced SDS-PAGE and were judged to be >95% pure. Protein identity was confirmed by western blotting using polyclonal antibody to mouse PrP. Protein concentration was determined using the Bio-Rad protein assay reagent. For each PrP preparation used, a dose-response curve of Plg activation was obtained as a reference for concentrations used in the assay. Concentrations of both wild type PrP and PrP mutant was calculated based on the molecular weight of 27 kDa, which meant 1mg/ml of PrP was equal to 37 μM

### Amyloid Fibrils

Amyloid fibrils were formed upon incubation of full-length recombinant mouse PrP (0.25 mg/ml, ~12 mM) at 37 °C in 3 M urea, 0.2 M NaCl, 10 mM sodium acetate buffer, pH 5.0, as described by Baskakov et al., (2002). The kinetics of fibril formation were monitored with a thioflavin T binding assay (LeVine, 1993). All were prepared at 2 M guanidine hydrochloride, 10 mM thiourea, 50 mM Mes, pH 6.0, at 37 °C with shaking and stored at 4 °C.

### Plg Activation Assays

The assays were performed at 37 °C in 96-well plastic microtitre plates containing 30 μl “assay buffer” (0.05 M Tris-HCl, 0.1 M NaCl [pH 7.4], 0.001% Tween 80), plus other components: these were tPA (0.25 nM) in the same Tris-Tween-NaCl buffer, and the plasmin-specific fluorogenic peptide substrate H-_D_-Val-Leu-Lys-7-amido-4-methylcoumarin (AMC; 0.2 mmol/L). Following 10 minutes incubation at 37 °C, the activation reaction was started by the addition of 12 nM Lys-Plg (diluted in the same assay buffer), maintaining a final total volume of 100 µl. Fluorescence intensity was measured continuously because of VLK-AMC hydrolysis at 37 °C, in a Spectra Max Gemini XS fluorescence spectrophotometer (Molecular Devices). In accordance with the optical characteristics of the fluorogenic group AMC, the following wavelengths were used: Absorption wavelength: λ_Abs._: 360 nm, emission wavelength: λ_Em._: 440 nm. Signals obtained were fed to a computer using SOFTmax pro software for storage, display, plotting, and manipulation.

### Data Analysis

The fluorescence data was exported from SOFTmax pro and converted into an EXCEL file. *δF/ δt* was calculated by plots of F versus *t*^2^. Rate of plasmin generation (V) was then calculated from the slope.

Samples were removed at time points and added to the fluorescence substrate. In this way the amount of plasmin generated in the original reaction was determined and plotted against time. Initially there is a continuous linear increase in plasmin generation but eventually all the Plg is converted to plasmin, with no further increase in plasmin generation, and the graph begins to plateau. Hence, for the initial part of the graph V = [Plasmin]/t. Using this equation along with the fact that dF/dt = [Plasmin], the equation 2(F/t^2^) = V was obtained, which means the rate of Plg activation can be determined from a graph of fluorescence against time squared and multiplying the value for the slope of the line by 2. This enabled quantification of the rate of Plg activation under different conditions.

### Kinetics of Plasminogen Activation

Kinetics of plasminogen activation were measured using the coupled enzymatic assay outlined above with slight modification. Briefly, individual assay samples encompassed the following components: 0.25 nM tPA, 1-500 nM Lys-Plg or 1-500 nM Glu-Plg. When Plg activation was analyzed in the presence of a cofactor, individual assays contained PrP or Desafib-X at varying concentration. The rate of Plg activation, ΔF/min, was plotted against the concentration of Plg. Kinetic parameter *K*_m_ and *V*_max_ were estimated from two-parameter best fits of the data to the Michaelis-Menten equation (*v = V*_*max*_*S/K*_*m*_* +S*) with use of a nonlinear curve fitting computer modeling program (Sigma Plot 10).

## Results

### PrP Increases the Activation Rate of Plg by tPA

The kinetics of the activation of Glu-Plg and Lys-Plg by two-chain tPA were studied in the absence or presence of PrP. This was compared to stimulation of tPA-catalyzed Plg activation by soluble fibrin fragment, Desafib-X (DFX). DFX has an advantage over insoluble fibrin leading to heterogeneous catalysis, which hampers the direct application of Michaelis-Menten kinetics.

The initial rate of activation for both types of Plg, in the absence of cofactor, did not approach saturation, hence it was not possible to calculate *K*_m_ and *V*_max_ values. However, the rate of plasmin generation was found to be 0.3 nM plasmin min^-1^ nM^-1^ for Lys-Plg, which was 8-fold greater than that measured for Glu-Plg (Table 1). This is because, in contrast to the compact form of native Plg, Lys^77^-Plg has an extended conformation so is more readily activated.

**Table 1:** Kinetic parameters of both native plasminogen Glu-Plg, and Lys-Plg, in the absence or presence of soluble fibrin, DFX (70 μg/ml), or prion protein, PrP (70 μg/ml or 3 μM)

	*K*_m_ (nM)	*V*_max_ (nM plasmin min^-1^)	*V*_max_/*K*_m_ (nM plasmin min^-1^ nM^-1^ Plg)
Lys-Plg – cofactor	–	–	0.3
Lys-Plg + DFX	12.5±1.43	98.0±1.79	7.84
Lys-Plg + PrP	42.1±10.50	332.1±20.41	7.89
Glu-Plg – cofactor	–	–	0.04
Glu-Plg + DFX	8.4±2.35	56.4±2.40	6.71
Glu-Plg + PrP	40.6±18.30	152±19.65	3.74

All assays were done in duplicate for each Plg concentration and were repeated at least 3 times. Mean ± SEM is shown.

**Fig. 1:**
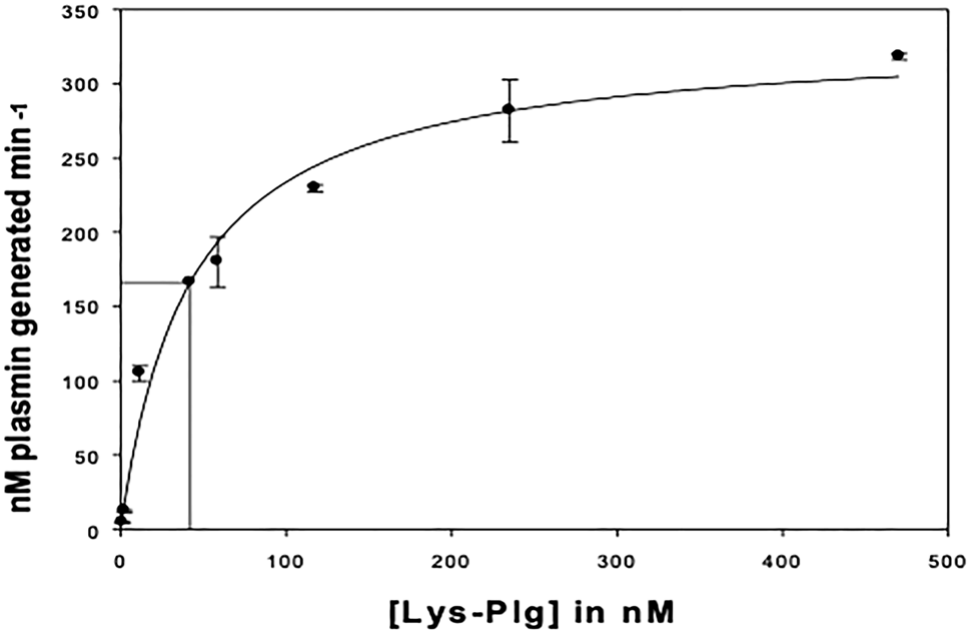
Investigating plasminogen (Plg) kinetics in presence of cofactor. Titration curves of Lys-Plg, activated by 0.25 nM tissue plasminogen activator in presence of 70 μg/ml prion protein. Mean ± SEM of two data sets is shown

In presence of PrP or DFX, Plg activation followed Michaelis-Menten kinetics, demonstrated by the fact that reactions now saturated, making it possible to determine the kinetic constants (Fig. 1).

Addition of PrP significantly increased the activation rate of Plg by tPA. The Michaelis constants and the catalytic rate constants of Plg activation at 70 μg/ml PrP (3 μM) for Glu-Plg (*K*_m_ = 40.6 nM and *V*_max_ = 152 nM plasmin min^-1^) and Lys-Plg (*K*_m_ = 42.1 nM and *V*_max_ = 332.1 nM plasmin min^-1^) indicate that in the presence of PrP, tPA has the same affinity for Lys-Plg and Glu-Plg, but that Lys-Plg is activated at twice the rate of Glu-Plg (Table 1). The second order rate constants (*V*_max_*/K*_m_; 3.74 and 7.89 nM plasmin min^-1^ nM^-1^ Glu-Plg and Lys-Plg, respectively) also reflect the higher substrate specificity for Lys-Plg than for Glu-Plg.

Like PrP, the Michaelis constant, *Km*, was the same for both forms of Plg (approximately 10 nM) in the presecnce of 70 μg/ml DFX (Table 1). The *V*_max_ for Glu-Plg activation by DFX-bound tPA was 56.4 nM plasmin min^-1^, and for Lys-Plg activation was found to be 98 nM plasmin min^-1^. However, unlike with PrP, there was no difference in the second order rate constants (*V*_max_*/K*_m_; 6.71 and 7.84 nM plasmin min^-1^ nM^-1^ for Glu-Plg and Lys-Plg, respectively) suggesting the mechanism by which PrP binds to Plg may be different to DFX.

### Differences Between PrP Isoforms

Given that copper, manganese, and nickel can bind PrP, the ability of PrP refolded with these cations to stimulate Plg activation, was tested. Increasing concentrations of recombinant PrP refolded with nickel, manganese or copper were added to tPA and Plg. The measured rate of Plg activation was expressed as fold stimulation, compared to the absence of PrP. In all cases bell shaped curves were obtained, indicating that PrP acts as a template regardless of the metal ion bound.

**Fig. 2:**
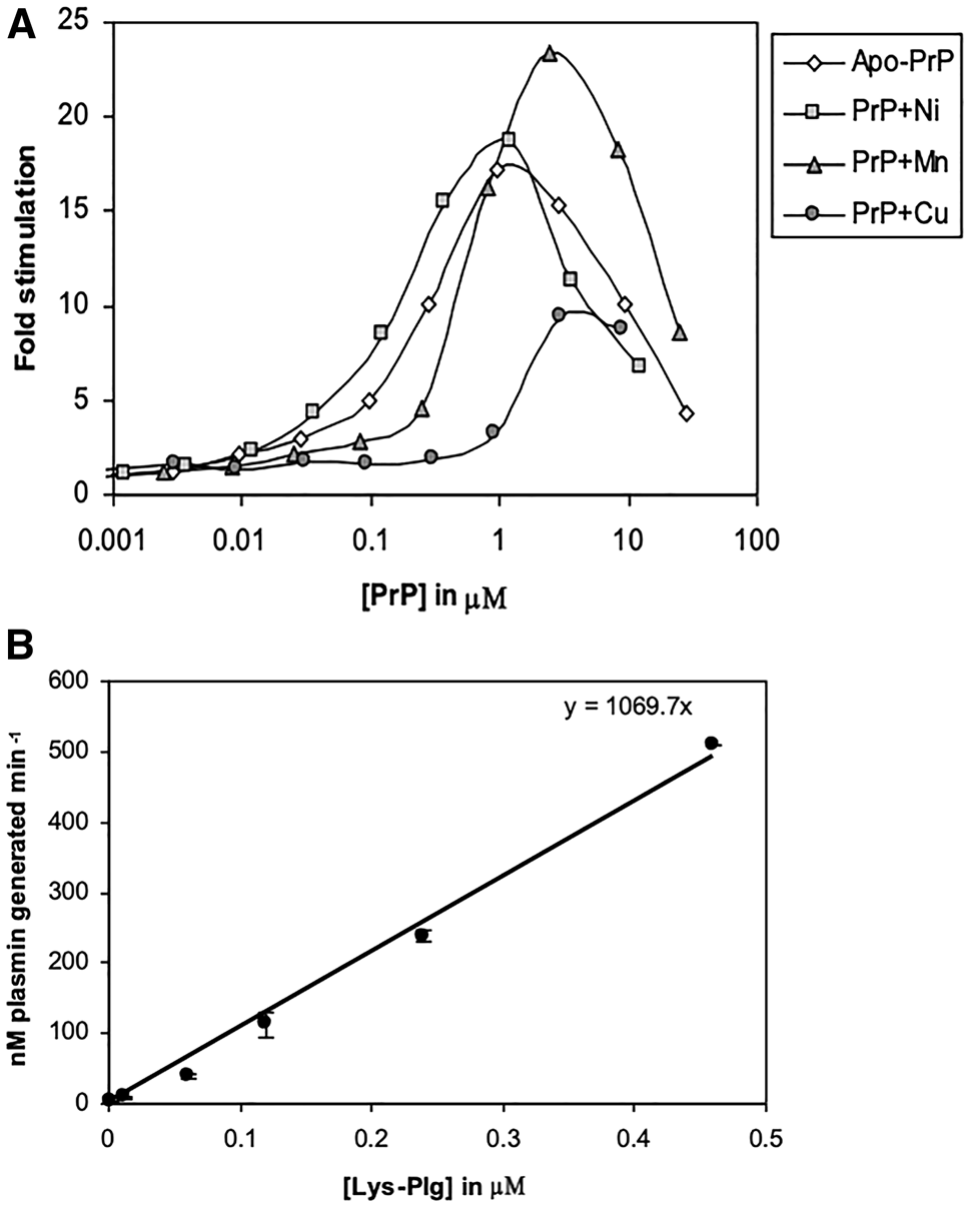
Comparison of effect of full length PrP refolded with various divalent cations on stimulation of Plg activation. **A** The effect of PrP refolded with different metal ions was assessed using the Plg activation assay. Increasing concentrations of PrP refolded with nickel (), manganese (), copper () or no metal ions () were added to individual assays. Values indicate the ability of the added protein to stimulate activation of Lys-Plg (12 nM). **B** Graph presenting Plg activation kinetics in presence of optimal concentration of PrP + Cu (3 μM). Mean ± SEM of two experiments is shown in B

However, data showed that the PrP isoforms behave differently relative to each other. As indicated in Fig. 1, apo-PrP (PrP devoid of bound metal) demonstrated substantial activity. PrP refolded with nickel (PrP+Ni) or manganese (PrP+Mn) showed similar activity to each other and to apo-PrP at 1 μM. Interestingly, the maximal stimulation observed with all three, was 5 times higher than that seen with PrP refolded with copper (PrP+Cu) (Fig. 2).

At the optimal concentration of 5 μM, PrP+Cu only produced a 10-fold increase in plasmin generation compared to almost 25-fold obtained with apo-PrP. It is clear from Fig. 2B that *K*_m_ is more than 0.5 μM for PrP+Cu, whereas based on results in Table 1, the *K*_m_ for apo-PrP is less than 50 nM. These findings suggest that copper-binding to PrP induces a change in conformation in the protein, converting it to a less effective cofactor.

### Effect of PrP Mutant on Plg Activation

Previously a mutant lacking the octameric repeat region (Δ51-90) was unable to stimulate Plg activation (Ellis et al., 2002), indicating that the octarepeat region is essential for efficient interaction of PrP with the Plg activation system. In this study, we aimed to find out if residues outside the region are also important in order for PrP to effectively stimulate Plg activation. A mutant, in which histidine residues outside the octarepeat region were mutated to alanine (H95A, H110A), was tested.

**Fig. 3:**
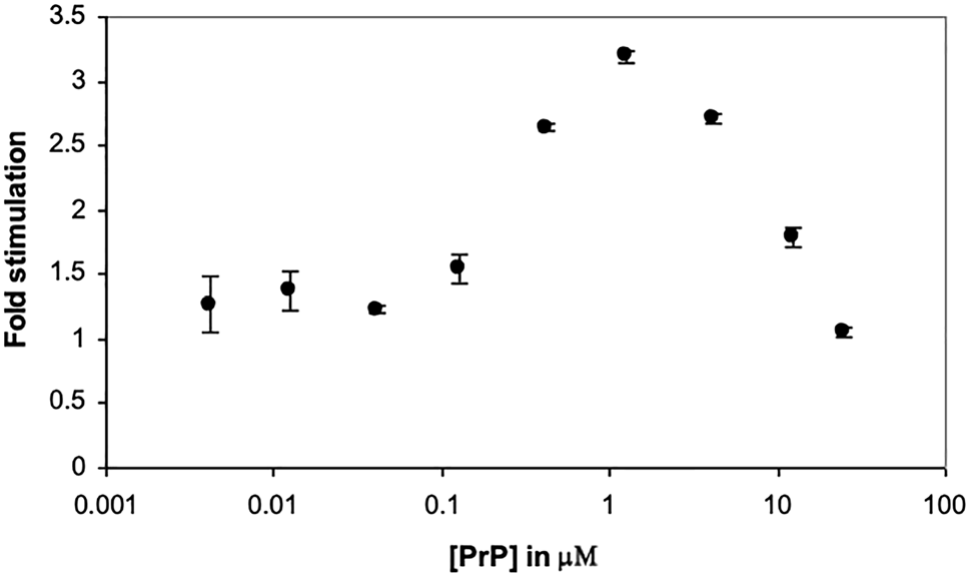
Stimulation of Plg activation by a PrP mutant. Increasing concentration of the mutant H95A, H110A (in which His residues at 95 and 110 were mutated to Ala) water refolded, was added to tPA and Plg. Fold stimulation was plotted against concentration. Mean ± SEM of two data sets is shown

When added to tPA and Plg, this mutant was unable to stimulate Plg activation as effectively as wild type PrP. H95A, H110A showed the same pattern of stimulation as wild-type PrP, but with a much lower maximal stimulation of 3-fold at 1 μM (Fig. 3). The *K*_m_ for Plg in the presence of H95A, H110A (8.3 nM) was similar to that with wild type PrP (10.4 nM, Table 2), indicating that the affinity of PrP for Plg is unaltered. However, *V*_max_/*K*_m_ was significantly lower with H95A, H110A (3.4 nM plasmin min^-1^ nM^-1^ Lys-Plg) suggesting much lower enzyme efficiency.

**Table 2:** Comparing plasminogen kinetics of mutated or aggregated prion protein (PrP) with wild type form

Preparation	*K*_m_ (nM)	*V*_max_ (nM plasmin generated min^-1^)	*V*_max_/ *K*_m_ (nM plasmin generated min^-1^ nM^-1^ Plg)
No PrP	–	–	0.6
Wild type PrP	10.4 ± 8.97	318.2 ± 52.62	30.6
H95A, H110A	8.3 ± 1.82	28.1 ± 1.11	3.4
Fibrils	21.3 ± 4.93	36.2 ± 1.99	1.7

Titration curves of Lys-Plg activated by 0.25 nM tPA, in the presence of wild type PrP (7 μg/ml, 0.3 μM), PrP mutant H95A, H110A (5 μg/ml, 0.2 μM) and fibrils (7.5 μg/ml) were obtained. For each one, mean and range of 3 sets of assays is shown. Mean ± SEM is shown

### PrP is an Effective Cofactor Only in the Monomeric Form

**Fig. 4:**
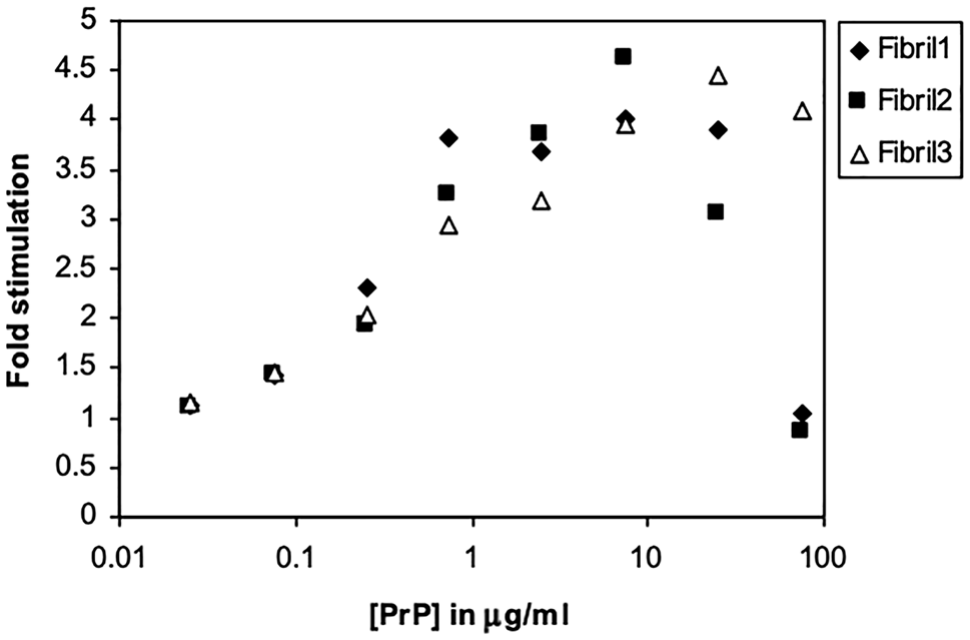
Stimulation of Plg activation by PrP fibrils. The samples differ from each other only by the time of their preparation and the protein stock used. They are all from full-length mouse PrP. The fibrils slowly undergo self-cleavage upon storage. Sample 3 had very little self-cleavage but samples 1 and 2 had some (~25%). Molecular weight of the protein is 23 kDa, self-cleavage products appear at 10-15 kDa

Recombinant mouse PrP produced in *Escherichia coli* was polymerized into amyloid fibrils. Three preparations of PrP fibrils were tested for their ability to stimulate Plg activation. Once again, a bell-shaped dose response curve was obtained but with maximal stimulation around 4-fold (Fig. 4). This suggests that PrP is an effective cofactor only in the monomeric form. When PrP aggregates, it loses the ability to potentiate Plg activation possibly because it interacts less well with tPA or Plg. Indeed, a slightly higher *K*_m_ was obtained for the fibrils compared to apo-PrP (Table 2).

## Discussion

In a continuous tPA-catalyzed Plg activation assay as used here, two reactions occur: Plg activation by tPA generates plasmin, which then cleaves the amide bond in VLK-AMC, generating the fluorogenic AMC. The rate of fluorogenic AMC production therefore reflects the rate of plasmin generation. In this study, two-chain tPA and Lys-Plg were used, since data analysis with the one-chain tPA derivative and native Plg would have been complicated, due to the feedback conversion by plasmin of one-chain tPA to two-chain tPA, and Glu-Plg to Lys-Plg.

The present study deals with a kinetic analysis of the activation of Lys-Plg by two-chain tPA, in the presence of PrP or DFX; a plasmin-digested fibrin fragment. PrP greatly stimulated tPA mediated Plg activation. No lag phase was observed in the plasmin generation curve, indicating that full length PrP is an effective stimulator of Plg activation.

In the purified systems containing PrP or DFX, Plg activation obeyed classical Michaelis-Menten kinetics. The derived kinetic parameters show that, in absence of cofactor, Plg is not activated efficiently. In the presence of cofactor however, the apparent Michaelis constant (*K*_m_) for both Glu-Plg and Lys-Plg is around 0.01 μM with DFX, and 0.04 μM with PrP. Both values are much lower than the plasma Plg concentration (about 1.5 μM), which indicates that tPA would efficiently convert Plg to plasmin in presence of DFX or PrP. PrP-mediated enhancement of catalytic efficiency resulted from 3-fold greater *V*_max_ than DFX.

Kinetic data suggest that fibrin binds tPA and Plg forming a ternary complex which results in a decrease of the *K*_m_ for Plg activation (Hoylaerts et al., 1982). The predominant effect of PrP, like that of fibrin, was a marked increase in *V*_max_/*K*_m_. This suggested that PrP also binds tPA and Plg to form a ternary complex, thereby increasing the local Plg concentration and reducing the apparent *K*_m_, through formation of a bridge between enzyme and substrate. Consistent with this, the stimulation was shown to display a bell-shaped curve with increasing PrP concentration (Fig. 2A). At higher concentrations of PrP, tPA and Plg become increasingly less likely to simultaneously interact with the same molecule of PrP, and the formation of PrP-tPA and PrP-Plg bimolecular complexes are favoured, over the formation of functional trimolecular complexes. This is consistent with a template model of catalysis.

However, the fact that *V*_max_/*K*_m_ with PrP was twice as high with Lys-Plg compared to Glu-Plg, whereas DFX stimulated activation of Glu- and Lys-Plg similarly , suggests a difference in binding mechanism between the two cofactors. Since PrP is only as effective as DFX when Lys-Plg is used, it suggests PrP may act as a cofactor of Plg stimulation only under certain conditions.

We demonstrated that there is a 10-fold decrease in Plg activation when PrP is bound to Cu^2+^, compared to apo-PrP (Fig. 2A). Since binding of nickel or manganese to PrP, did not produce a corresponding decrease in activity, suggests they may not cause the same structural change in PrP compared to copper. It is likely that the decrease in activity seen with PrP+Cu^2+^ results from a structural rearrangement that masks the binding sites for tPA and/or Plg. Thus, the ability of PrP to bind copper, and the ability to stimulate Plg activation are inversely related.

Here, a mutant of mouse recombinant PrP was obtained, in which the histidine residues at 95 and 110 had been mutated to alanine. The mutant was unable to stimulate Plg activation as efficiently as wild type PrP (Fig. 3). Therefore, histidine residues in the region 90-120 are also essential for maintaining a form of PrP that can efficiently interact with tPA and Plg. Preventing the function of these residues, either by copper binding or mutation, will most likely result in a change in PrP structure and hence function. It may be that interaction of copper with the histidine residues around the plasmin cleavage site (residues 95 and 110) masks the site, reducing the chance of cleavage by plasmin. Functionally, this may suggest that the region between the N-terminal octarepeat domain and the structured C-terminal domain acts as a switch. Copper binding to His96/111 could prepare the protein for endocytosis, while at lower concentrations of copper ions, PrP is cleaved, releasing the N-terminal fragment. Indeed, binding of extracellular Cu^2+^ ions to the octarepeat region have been shown to stimulate PrP^C^ endocytosis (Pauly & Harris, 1998, Brown & Harris, 2003), abrogated by alterations to the number of octapeptide repeats (Krasemann et al., 1995, Perera & Hooper, 2001). Alternatively, coordination of Cu^2+^ ion to both His96 and His111 may induce a β-sheet like conformation (Jones et al., 2004). Jobling et al., reported that aggregation of a neurotoxic PrP-derived peptide (106-126) is increased by the binding of copper ions (Jobling et al., 2001). Another possibility is that changes in the secondary structure of PrP^C^, when converted to PrP^Sc^, cause the toxic domain to be unmasked, which is not cleaved by plasmin due to the bound copper ion.

Fibrils prepared by polymerization of full-length recombinant mouse PrP were also tested for their ability to stimulate Plg activation. The fibrils behaved similar to the H95A, H110A mutant, with ~ 4-fold stimulation at the optimal concentration compared to the absence of cofactor (Fig. 4), possibly reflecting the inability of the proteins to form stable complexes with both tPA and Plg. However, it must be noted that *in vitro,* recombinant PrP fibrils may behave differently to PrP^sc^ and thus may not be truly representative.

From the data presented in this study, it is clear that PrP is able to act as an efficient promoter of Plg activation, by the soluble extracellular protease; tPA. The level of stimulation was up to 25-fold, which although less than that observed in previous studies with PrP (Ellis et al., 2002), it is still a significant finding. This stimulatory effect involves a mechanism in which PrP acts to provide a specific template for the assembly of a ternary complex involving tPA and Plg.

Whilst bound to copper, PrP weakly stimulates Plg activation. Yet in the absence of copper, PrP becomes an efficient cofactor, but only if Lys-Plg is present. The closed conformation of Glu-Plg means it still interacts with PrP but is activated to a less degree, possibly due to less chance of ternary complex formation with tPA. Consequently Glu-Plg may act as an inhibitor of PrP endocytosis and promote its conversion to PrP^sc^ (Mays & Ryou, 2011). This may be why in the spleen of Plg deficient mice, significantly less PrP^Sc^ is seen (Salmona et al., 2005). It may be that in prion disease, either, raised copper ion concentrations causes the rate of copper binding and misfolding to exceed rate of PrP endocytosis, or Plg activation prior to PrP interaction is disrupted, leading to protease resistance and aggregation.

## Data Availability

All relevant data are contained within the manuscript.

## Abbreviations

PrP: Prion Protein
Plg: Plasminogen
tPA: Tissue Plasminogen Activator
Kr: Kringle
uPA: Urokinase Plasminogen Activator
ECM: Extracellular Matrix
Abeta: Amyloid-beta
DFX: Desafib-X
